# A p53-independent apoptotic mechanism of adenoviral mutant E1A was involved in its selective antitumor activity for human cancer

**DOI:** 10.18632/oncotarget.10221

**Published:** 2016-06-22

**Authors:** Lin Fang, Qian Cheng, Jingjing Zhao, Yan Ge, Qi Zhu, Min Zhao, Jie Zhang, Qi Zhang, Liantao Li, Junjie Liu, Junnian Zheng

**Affiliations:** ^1^ Jiangsu Key Laboratory of Biological Cancer Therapy, Xuzhou Medical University, Xuzhou, Jiangsu Province, 221002, China; ^2^ Affiliated Hospital of Taishan Medical University, Taian, Shandong Province, 271000, China; ^3^ Cancer Center, Affiliated Hospital of Xuzhou Medical University, Xuzhou, Jiangsu Province, 221002, China; ^4^ Jiangsu Center for the Collaboration and Innovation of Cancer Biotherapy, Xuzhou Medical University, Xuzhou, Jiangsu Province, 221002, China

**Keywords:** adenoviral E1A, Rb, p53, cell apoptosis, hepatocellular carcinoma

## Abstract

The conserved regions (CR) of adenoviral E1A had been shown to be necessary for disruption of pRb-E2F transcription factor complexes and induction of the S phase. Here we constructed a mutant adenoviral E1A with Rb-binding ability absent (E1A 30-60aa and 120-127aa deletion, mE1A) and investigated its antitumor capacities *in vitro* and *in vivo*. The mE1A suppressed the viability of tumor cells as efficiently as the wild type E1A, and there was no cytotoxic effect on normal cells. Although the mE1A arrested tumor cell cycle with the same manner as E1A, the former played a different role on cell cycle regulation compared with E1A in normal cells, which might contribute to its selective antitumor activity. E1A and mE1A had accumulated inactive p53, decreased the expression of mdm2, Cdkn1a (also named p21), increased p21's nuclear distribution and induced tumor cell apoptosis in a p53-indenpent manner. Further, E1A or mE1A significantly suppressed tumor growth in subcutaneous hepatocellular carcinoma xenograft models. Especially, tumor-bearing mice treated with mE1A had higher survival rate than those treated with E1A. Our data demonstrated that mutant adenoviral E1A significantly induced tumor cell apoptosis in a p53-indenpednt manner and had selective tumor suppressing ability. The observations of adenoviral E1A mutant had provided a novel mechanism for E1A's complex activities during infection.

## INTRODUCTION

The human adenoviral early region 1A (E1A) is the first viral protein to be expressed following infection [1]. It was originally considered as an oncogene of the adenovirus genome [2]. Intriguingly, E1A is identified and associated with multiple anti-cancer activities by modulating host cell transcriptional machinery [3–7]. It has been reported that E1A induced cell apoptosis in human cancer cell lines *in vitro* and *in vivo* and suppressed cancer metastasis [8, 9]. The clinical trials with E1A/liposome gene therapy for breast, ovarian and head/neck cancers had been conducted [10–12]. However, the molecular mechanism mediated by adenoviral E1A for this tumor inhibition is not yet clearly described.

E1A alternative splicing generates five mRNAs and two major products are the 13s and 12s mRNAs, which encode proteins of 289 and 243 amino acids respectively. There are four conserved regions (CR1-CR4) within 13S-encoded protein [13]. E1A has no enzymatic or specific DNA binding capabilities and carries out its functions by interacting with a large number of cellular proteins through its linear motifs within the CR regions, e.g. with the cellular transcription factors TATA-binding protein (TBP), c-Jun, the co-activator p300/CBP and the cell cycle inhibitors pRb and p21 [8–14]. The first identified protein interacted with adenoviral E1A was retinoblastoma tumor suppressor protein (pRb) which is a key regulator of cell cycle progression [15]. Adenoviral E1A disrupted the interaction between pRb and E2F, allowing transactivation of genes necessary for viral DNA replication [16, 17]. Two E1A conserved regions are responsible for this disruption, CR1 and CR2. In order to preserve the antitumor function of pRb, there had been reported that E1A CR2 deleted adenovirus dl922-947 has considerable activity in ovarian cancer and induces cell death through a non-apoptotic mechanism [18].

The tumor suppressor p53 (Trp53) was initially identified and characterized by its interactions with viral oncoproteins; one of them is the adenoviral E1B 55kd protein. E1B 55kd inhibits p53-meidated transcriptional activation. However, it had been reported that E1B 55kd did not affect cellular response during infection in cancer cells with mutated or null p53 status [19]. The other possible pathway of p53 regulation was needed to investigate upon adenovirus infecting. Although there is research about E1A had been involved in p53 regulation [8–20], the understanding of E1A mediated tumor suppression activity remains limited.

In this study, we have constructed a mutant adenoviral E1A, deletion of portion CR1 and CR2 (CR1, 30-60aa and CR2, 120-127aa), and identified its selective antitumor activity compared to wild type E1A. We showed that E1A and mE1A had accumulated inactive p53, decreased the expression of p53 target proteins (p21 and mdm2). E1A and mE1A had induced tumor cell apoptosis in a p53-indenpent way. The cellular p21 protein and its nuclear distribution after infected with Ad-DC315-E1A or Ad-DC315-mE1A was responsible for adenoviral E1A mediated antitumor activity. Further, Ad-DC315-E1A or Ad-DC315-mE1A inhibited tumor growth in subcutaneous hepatocellular carcinoma cell xenograft models. Tumor-bearing mice treated with Ad-DC315-mE1A had higher survival rate than those treated with Ad-DC315-E1A. The observations of adenoviral E1A mutant had provided a novel mechanism for E1A's complex activities during infection.

## RESULTS

### The mutant E1A (mE1A) was lack of cellular Rb binding ability

**Figure 1:**
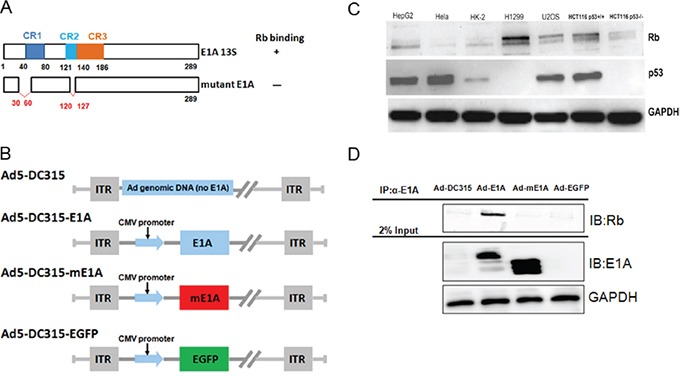
The mutant adenoviral E1A lost the ability to bind to cellular Rb protein **A.** Schematic representation of adenoviral E1A 13s and the deletion mutant E1A structures. mE1A: mutant E1A with deletion 30-60aa and 120-127aa. **B.** Schematic diagrams of Ad-DC315, Ad-DC315-E1A, Ad-DC315-mE1A and Ad-DC315-EGFP structures. **C.** Hela, HepG2, HK-2, H1299, U2OS, HCT116 p53+/+ and HCT116 p53−/− cells were lysed and lysates were to detect Rb and p53 expression by western blotting. GAPDH served as the protein loading control. **D.** HepG2 cells were infected with Ad-DC315, Ad-DC315-EGFP, Ad-DC315-E1A and Ad-DC315-mE1A at MOI=20. Cells were lysed at 48 hours post infection and subjected to immunoprecipitation with anti-E1A antibody. Protein Rb was detected by immunoblotting analysis (IB).

We firstly detected the Rb and p53 protein expression levels in the tumor cell lines and HK-2 cells. As shown in Figure 1C, there was various expression of Rb in the tested cell lines. There were high Rb in HepG2, H1299 and U2OS cells. The p53 level was high in HepG2, Hela, U2OS and HCT116p53+/+ cells. As expected, p53 was null in H1299 and HCT116p53−/− cells. We then examined the express of E1A (mE1A) protein mediated by adenovirus in HepG2 cancer cells using western blot analysis. As shown in Figure 1D, the Ad5-DC315-mE1A expressed E1A protein was smaller than Ad5-DC315-E1A because of the deletion of 30-60 and 120-127aa. Wild type E1A itself could bind to cellular Rb protein and inhibited the tumor suppressor ability of Rb. The constituted mE1A was designed to protect the Rb function. Then we preformed immunoprecipitation (IP) assay to detect whether mE1A could bind to Rb. HepG2 cells were infected with Ad-DC315, Ad-DC315-EGFP, Ad-DC315-E1A or Ad-DC315-mE1A at MOI=20 for 48h. Total cell lysates were harvested for IP. The data showed that there was an Rb band under Ad-DC315-E1A treatment, but there was no band for other treatments. These results suggested that mutant E1A (mE1A), unlike wild type E1A, had lost the ability to bind to Rb protein.

### E1A/mE1A had tumor suppressing ability

**Figure 2:**
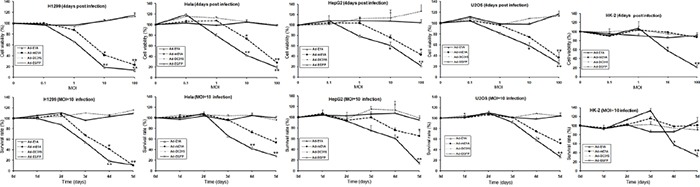
Ad-DC315-mE1A has antitumor activity against human cancer cells but not normal cells H1299, Hela, HepG2, U2OS, HK2 cells were infected with Ad-DC315, Ad-DC315-E1A, Ad-DC315-mE1A or Ad-DC315-EGFP at the indicated dose (0.1, 1, 10,100) for 4 days or for the indicated days (1-5 days) at MOI=10. Cell viability was measured by MTT assay. The value of MOI=0 or day 1 was set at 1. Cell viability data was expressed as mean values ±SD (n=6). Statistical significance was determined using Student's *t* test. *P<0.05, ** *P*<0.01.

To investigate the *in vitro* antitumor activity of Ad-DC315-E1A or Ad-DC315-mE1A, cell viability was measured using the MTT assay. H1299, Hela, HepG2, U2OS and HK-2 cells were infected with Ad-DC315, Ad-DC315-EGFP, Ad-DC315-E1A or Ad-DC315-mE1A at the indicated dose (0.1, 1, 10,100) for 4 days or for the indicated days (1-5 days) at MOI=10. The data showed that Ad-DC315-E1A and Ad-DC315-mE1A inhibited tumor cell proliferation in a dose and time-dependent manner. Ad-DC315-E1A, Ad-DC315-mE1A inhibited cancer cells more efficiently than the controls (Ad-DC315 or Ad-DC315-EGFP) (Figure 2). Moreover, Ad-DC315-E1A induced a litter more tumor inhibiting ability than Ad-DC315-mE1A. However, Ad-DC315-E1A could cause normal cells (HK-2) death even at a low MOI (10) compared to Ad-DC315 or Ad-DC315-EGFP, while Ad-DC315-mE1A had no effect to normal cells even at high MOIs, which acted similarly to Ad-DC315 or Ad-DC315-EGFP.

### E1A/mE1A affected cell cycle distribution

**Figure 3:**
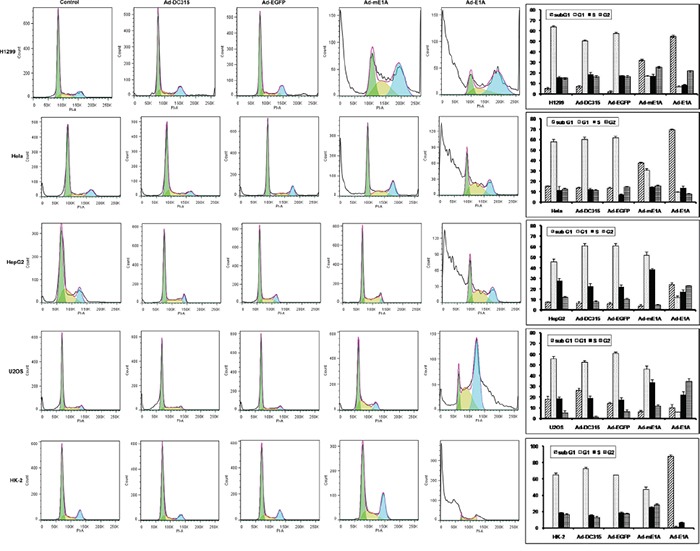
Ad-DC315-mE1A or Ad-DC315-E1A affected cell cycle distribution in different manners H1299, Hela, HepG2 and U2OS cells were infected with Ad-DC315, Ad-DC315-E1A, Ad-DC315-mE1A or Ad-DC315-EGFP at MOI=20 for 72h. The cell cycle was assessed using flow cytometry analysis. Three independent trials were performed and the mean value was shown on the right.

Adenovirus entering into host cells forces the latter into cell cycle, and the E1A protein plays a critical role during the process. E1A is the initiator and primary executor of the cell cycle modulation. Then we want to determine the direct role of E1A or mE1A on host cell cycle regulation. As shown in Figure 3, Ad-DC315-E1A and Ad-DC315-mE1A had a significant increased subG1 phase in infected H1299 and Hela cells. In HepG2 and U2OS cells, Ad-DC315-E1A and Ad-DC315-mE1A were found to induce S/G2 phase arrest compared to the control groups. Intriguingly, Ad-DC315-E1A induced dramatically more subG1 phase in normal HK-2 cells than the controls, but not for Ad-DC315-mE1A, and Ad-DC315-mE1A acted very similarly to the Ad-DC315 or Ad-DC315-EGFP treatment group. These data indicated that Ad-DC315-mE1A regulated tumor cell cycle with the same manner with Ad-DC315-E1A, but in normal cells Ad-DC315-mE1A played a different role on cell cycle regulation compared with Ad-DC315-E1A, which might contribute to its selective antitumor activity.

### E1A/mE1A induced cell apoptosis in a p53-independent manner

**Figure 4:**
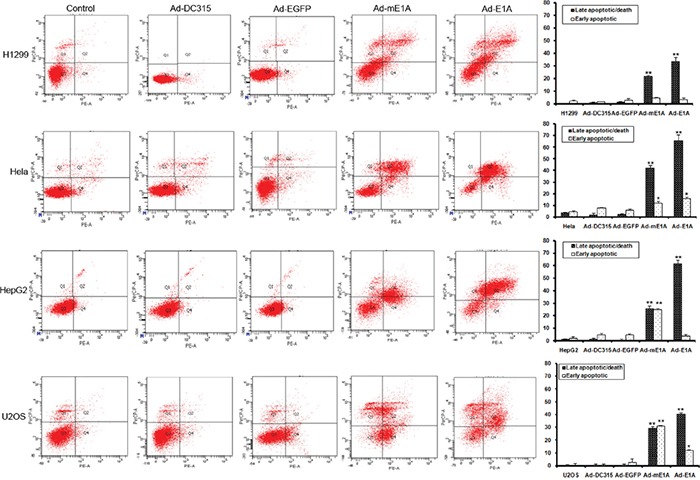
Ad-DC315-mE1A or Ad-DC315-E1A induced apoptosis in a p53-independent manner HCT116 p53+/+, HCT116 p53−/−, H1299, Hela, HepG2 and U2OS cells were infected with Ad-DC315, Ad-DC315-E1A, Ad-DC315-mE1A or Ad-DC315-EGFP at MOI=20 for 72h. The early and late stage apoptosis was quantified using flow cytometry analysis. Representive data was shown. Statistical significance was determined using Student's *t* test. *P<0.05, ** *P*<0.01.

E1A had been reported involved in cancer cell apoptosis processing during adenoviral infection. In order to determine whether the mE1A had this function, HCT116 p53+/+, HCT116 p53−/−, HeLa (p53-defective), H1299 (p53-null), U2OS (p53 wild type) and HepG2 (p53 wild type) cells were infected with Ad-DC315, Ad-DC315-E1A, Ad-DC315-mE1A or Ad-DC315-EGFP at MOI=20 for 72h. The early and late stage apoptosis was quantified using flow cytometry analysis (Figure 4). In this context, Ad-DC315-E1A and Ad-DC315-mE1A efficiently increased the percentage of apoptotic cells, although the apoptosis rate mediated by Ad-DC315-mE1A was a little less than that meditated by Ad-DC315-E1A. More interesting, Ad-DC315-mE1A seemed induced much more early apoptotic cells in HepG2 and U2OS cells, which obtained a wild type p53 status, but not in H1299 (p53 null) and Hela (defective p53). There was no or less apoptosis induced after Ad-DC315 or Ad-DC315-EGFP infection. Further, we found that in p53-null H1299 and HCT116 p53−/−cells, Ad-DC315-E1A orAd-DC315-mE1A also trigged cell apoptosis. These data confirmed that Ad-DC315-E1A or Ad-DC315-mE1A was sufficient to induce apoptosis, which acted in a p53-independent manner.

**Figure 5:**
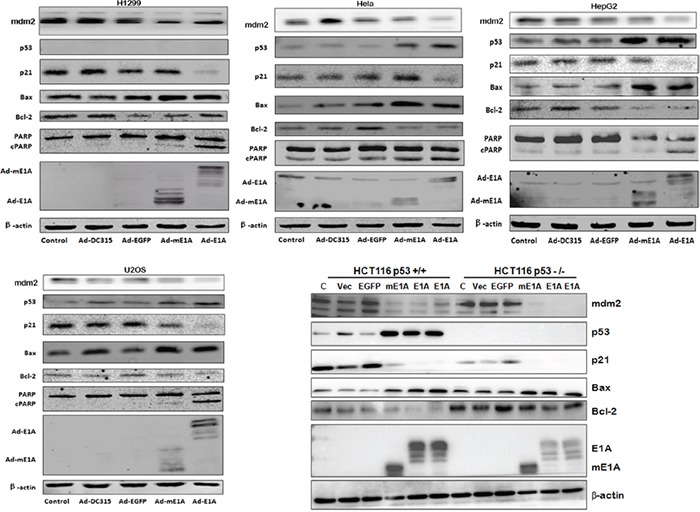
Ad-DC315-mE1A or Ad-DC315-E1A induced inactive p53 expression HCT116 p53+/+, HCT116 p53−/−, H1299, Hela, HepG2 and U2OS cells were infected with Ad-DC315, Ad-DC315-E1A, Ad-DC315-mE1A or Ad-DC315-EGFP at MOI=20 for 48h. The level of p53, p21, mdm2, Bax, Bcl-2, PARP and E1A proteins were analysed by western blotting. Beta-actin was assayed as a loading control.

Tumor suppressor p53 is well known a critical protein involved in apoptosis. To investigate the molecular mechanism of E1A/mE1A induced cell apoptosis, the apoptosis-associated proteins were detected by western blot (Figure 5). Ad-DC315-E1A or Ad-DC315-mE1A infection induced more p53 expression than the controls, exception of H1299 cells (p53-null) and HCT116 p53−/− cells. The p53-downstream target proteins such as p21, Bax and mdm2 were also evaluated. The expression of p21 and mdm2 was down regulated despite of high level of p53 protein. Increased Bax, cleaved parp (c-parp) and decreased bcl-2 proteins were also detected. These results suggested that E1A or mE1A mediated by Ad-DC315-E1A or Ad-DC315-mE1A stabilized p53, but the accumulated p53 was inactivated since it did not increase the amount of its targeted gene products. It also indicated that Ad-DC315-E1A or Ad-DC315-mE1A could accumulate inactivated p53, down regulated p21 and mdm2 expression and did induce tumor cell apoptosis in a p53-independent manner.

### Protein p21expression was involved in E1A or mE1A mediated apoptosis

**Figure 6:**
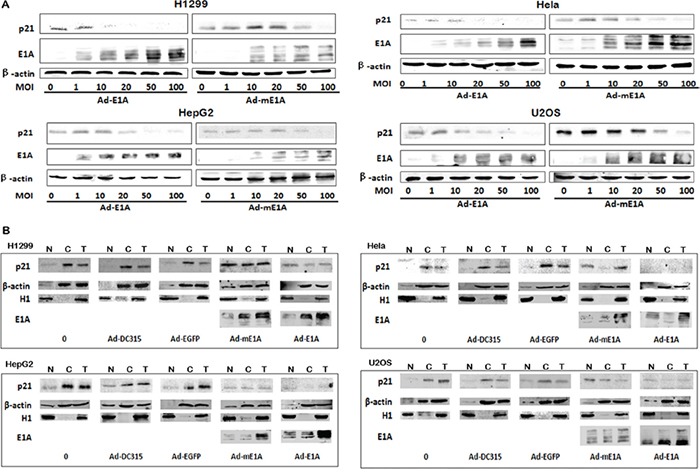
Ad-DC315-mE1A or Ad-DC315-E1A affected cellular p21 localization **A.** H1299, HeLa, U2OS and HepG2 cells were infected with Ad-DC315-E1A or Ad-DC315-mE1A at indicated MOIs for 72h. p21 and E1A levels were determined by immunoblot analysis. Beta-actin was used as a loading control. **B.** H1299, HeLa, U2OS and HepG2 cells were treated with adenoviruses at a MOI=20 for 48h. Cellular fractionation was performed. The p21 was detected in cytoplasmic and nuclear fraction (N, nuclear fraction; C, cytoplasmic fraction; T, total cell lysates.). H1 was used as a nuclear marker and btea-actin was a cytoplasm marker.

The cellular p21 (a cyclin-dependent kinase inhibitor) is critical for cell cycle regulation. The above data showed p21 was down regulated by E1A. We want to know whether this decreased p21 might be related to the E1A (mE1A)-mediated cell apoptosis and antitumor activity. H1299, HeLa, U2OS and HepG2 cells were infected with Ad-DC315-E1A or Ad-DC315-mE1A at MOIs of 1, 10, 20, 50 or 100 for 72h. Total cell lysates were harvested for western blot assay. As shown in Figure 6A, Ad-DC315-E1A and Ad-DC315-mE1A downregulated endogenous p21 expression in a dose-dependent manner in these tumor cells.

E1A is known to alter the subcellular distribution of proteins in order to alter their function. Then the sub-cellular fractionation of p21 was performed to determine its localization in cells infected with Ad-DC315, Ad-DC315-EGFP, Ad-DC315-E1A or Ad-DC315-mE1A. The p21 protein was largely cytoplasmic in cultured cells and cells infected with Ad-DC315 or Ad-DC315-EGFP (Figure 6B), and p21 might be targeted for proteasomal destruction. Intriguingly, the p21 transferred much more to the nucleus than that to the cytoplasma in cells infected with Ad-DC315-E1A or Ad-DC315-mE1A compared to the other treatments, although E1A or mE1A decreased the p21 expression level.

**Figure 7:**
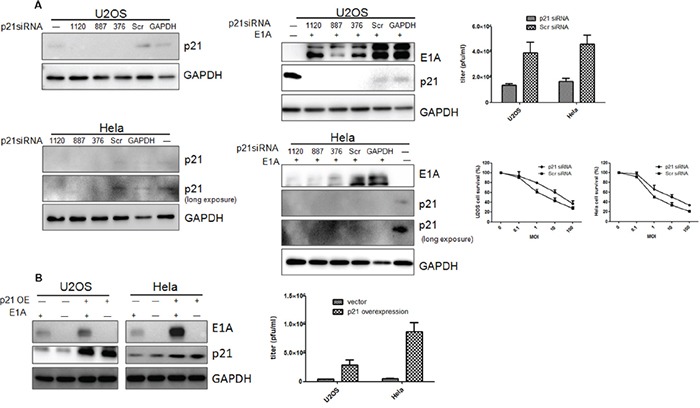
Protein p21expression was involved in E1A or mE1A mediated apoptosis **A.** 3 siRNA (1120nt, 887nt and 376nt) targeted for p21was transfected into U2OS and Hela cells, respectively. Src, scrambled siRNA. GAPDH, a siRNA construct targeted for cellular GAPDH gene, was used as a positive control for detecting siRNA efficiency. Ad-DC315-E1A was added into cells tranfected with p21or control siRNAs post 24h. E1A, p21 or GAPDH were detected by western blotting. The adenoviral infectious virion production was assessed 48h post infection by TCID50 (upper, right). Cell survival was analyzed 96h later by MTT assay in U2OS and Hela cells (bottom, right). **B.** The p21stablely-overexpressed U2OS and Hela cells were infected with Ad-DC315-E1A for 48h. The p21 and E1A proteins were detected. The adenoviral infectious virion production on p21-overexpressed cells was assessed 48h post infection by TCID50 (right).

Whether the p21 nuclear localization after Ad-DC315-E1A treatment contributed to the cell apoptosis, we next examined the cell response in cells infected with Ad-DC315-E1A followed p21 knockdown or overexpression. Utlizing 3 siRNA (target for p21 1120nt, 887nt, 376nt, designed and synthesized by GenePharma comapny, China) constructs, p21 knockdown was determined in U2OS and Hela cells 48 hours post transfection. The p21 knockdown mediated by siRNA reduced E1A expression and infectious virion production (Figure 7A). Cell cytotoxicity was also assessed in p21-knockdown cells followed by infecting with Ad-DC315-E1A for 96 hours. As shown in Figure 7A (bottom right), there was significantly less cell death in the p21 knockdown cells than that treated with scrambled siRNA. Next, we constructed stable p21-overexpressed U2OS and Hela cell lines using letivirus Lenti-X™ HTX Packaging System (Clontech, USA). The data showed that the cells overexpressed p21 had enhanced E1A expression and increased production of virions (Figure 7B). Together, these data indicated that p21 expression in cells was associated with adenoviral E1A mediated antitumor activity.

### Antitumor efficacy of Ad-DC315-E1A and Ad-DC315-mE1A in tumor xenograft nude mice

**Figure 8:**
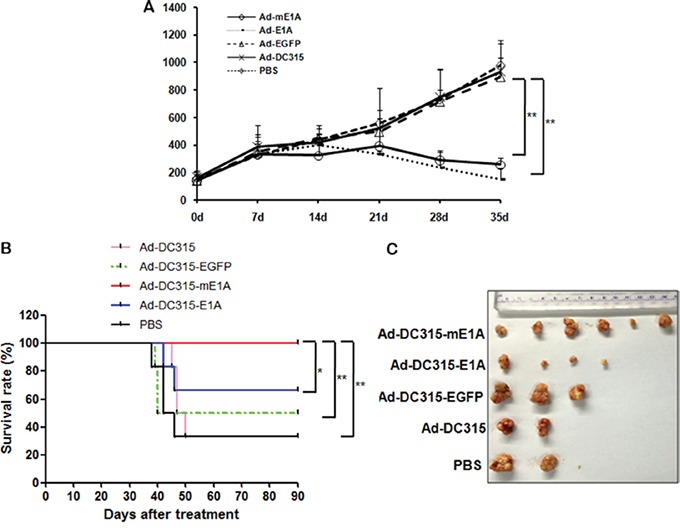
The antitumor efficacy of Ad-DC315-E1A and Ad-DC315-mE1A in tumor xenograft nude mice **A.** HepG2 cells (5 × 10^6^ cells per site) were inoculated into the flank of 5-week-old female BALB/c nu/nu mice. When the tumors reached 3-5 mm in diameter, Ad-DC315, Ad-DC315-E1A, Ad-DC315-mE1A, Ad-DC315-EGFP or PBS was intratumorally injected on days 0, 2 and 4. Tumour growth is expressed as the mean tumor volume ± SD in each group (n = 6). Statistical significance was determined using Student's t test. *P<0.05, ** *P*<0.01. **B.** Survival rate in each group of HepG2 tumors-bearing mice (n = 6) was shown using the Kaplan–Meier method. Statistical significance was determined using log-rank test. *P<0.05, ** *P*<0.01. **C.** The tumor mass removed from survival mice was shown.

Finally, the antitumor effects of Ad-DC315-E1A or Ad-DC315-mE1A in a human cancer cell HepG2 xenograft model were examined. HepG2 cells (2×10^6^) were subcutaneously inoculated into the right flank of 4 weeks-old female nude mice obtained from the Institute of Animal Center (Chinese Academy of Sciences, Shanghai). Animal welfare and experimental procedures were carried out strictly in accordance with the Guide for the Care and Use of Laboratory Animals. About when tumor diameters reached about 5-8 mm, mice were randomly divided into five groups (six mice per group) and treated by intratumor injections of Ad-DC315, Ad-DC315-E1A, Ad-DC315-mE1A or Ad-DC315-EGFP at a dose of 7×10^8^ plaque forming unit (PFU) per mouse every other day for three times or with PBS as a control. During the therapeutic days, tumor size was measured every 7 days. Within 35 days post the first treatment, there was no mouse dead. The data of tumor volumes showed that Ad-DC315-E1A or Ad-DC315-mE1A administration significantly suppressed tumor growth compared to Ad-DC315, Ad-DC315-EGFP or PBS groups (Figure 8A). Furthermore, the survival rate of mice with tumor was assessed until 90 days after first treatment. The tumor-bearing mice treated with Ad-DC315-mE1A had higher survival rate than other treatment (Figure 8B). The survival mice were sacrificed on day 90 and the tumors were removed and imaged (Figure 8C). These data suggested that Ad-DC315-mE1A eliminated tumor efficiently and was safer than Ad-DC315-E1A for nude mice.

## DISCUSSION

The previous work showed that adenoviral E1A with a CR2 portion deletion downregulated cell membrane receptor Her-2/neu expression and inhibited tumor progression in hepatocarcinoma xenografts model [21, 22]. The truncated minimal E1A acts as a tumor inhibitor gene. In this present work, we demonstrated that a mutant adenoviral E1A (mE1A) deletion of portion CR1 and CR2 (30-60aa and 120-127aa) can selectively inhibit tumor cell growth, but had no cytotoxic effect on normal cells, while the wild type E1A induced normal cells death dramatically (Figure 2). It had been reported that the adenoviral E1A CR2 region binds with high affinity to the B-domain of the pRb pocket whilst the CR1 region displaces E2F from the E1A CR2/pRb complex by low affinity binding with pRb directly at the E2F binding site [23]. The mE1A here we constructed was determined losing the ability of binding to Rb completely (Figure 1C). The cellular pRb had been preserved by this E1A deletion modification while retaining an unimpaired antitumor activity of E1A.

E1A induced tumor cell apoptosis in a p53-dependent manner [5]. It increased sensitivity to different categories of anticancer drug in multiple clinical trials [20]. Here we identified that this mE1A affected tumor cell cycle distribution in a different way as that in normal cells and induced cell apoptosis in a p53-independent manner (Figures 4&5). As known, there is different p53 status in various type of cancer. It had been reported the cellular mutant p53 exerted oncogenic gain-of-function (GOF) properties, but the mechanisms mediating these functions remain poorly defined [24, 25]. E1A regulates a multitude of cellular proteins. We described here the adenoviral E1A or mE1A had accumulated inactive p53, since p53-downstream target proteins p21 and mdm2 decreased while there was high p53 expression level. E1A proteins had been identified they did not form detectable complexes with p53 [26, 27]. It seemed that E1A could stabilize p53 protein, but in an inactivated form through an unclear mechanism.

In response to genotoxic stress, the cyclin-dependent kinase inhibitor p21 translocaes to the nucleus where it cause cell cycle arrest and promotes nucleotide excision repair following association with PCNA [28]. In our work, we showed the decreased p21 protein level and its nucleular distribution after infected with Ad-DC315-E1A or Ad-DC315-mE1A (Figure 6A&6B). The p21 knockdown mediated by siRNA reduced E1A expression and infectious virion production (Figure 7A). Cell cytotoxicity assay results suggested that there was significantly less cell death in the p21 knockdown cells than that treated with scrambled siRNA. The cells overexpressed p21 had enhanced E1A expression and increased production of virions (Figure 7B). These data indicated that p21 expression was associated with adenoviral E1A mediated antitumor activity. Although there were publications showed that p21 expression reduced oncolytic adenovirus activity in tumor cells [29], our data was identical with the reports that p21 overexpression appeared an important factor in elevating anti-tumor activity mediated by adenoviral E1A [30].

E1A mediated antitumor functions *in vitro* provided a rationale in animal experiment. Further, Ad-DC315-E1A or Ad-DC315-mE1A significantly suppressed tumor growth in subcutaneous hepatocellular carcinoma cell xenograft models (Figure 8). Tumor-bearing mice treated with Ad-DC315-mE1A had higher survival rate than those treated with Ad-DC315-E1A. Our data demonstrated that adenoviral E1A with 30-60aa and 120-127aa deletion significantly induced tumor cell apoptosis in a p53-indenpednt manner and had selective tumor suppressing ability. The observations of adenoviral E1A mutant had provided a novel mechanism for E1A's complex activities during infection.

In conclusion, we had clearly demonstrated that adenoviral E1A induced cell apoptosis in a p53-independent way. The adenoviral mutant E1A had an equal antitumor activity with wild type E1A, but a much safer one for administration deliver.

## MATERIALS AND METHODS

### Cell lines and plasmids

Human cell lines HeLa (p53-defective), H1299 (p53-null), HCT116 p53+/+, HCT116 p53−/−, U2OS (p53 wild type), HepG2 (p53 wild type), HEK (human embryonic kidney)-293(expressing E1A, p53 wild-type), human renal tubular epithelial cell HK-2 were maintained in DMEM (Dulbecco's modified Eagle's medium) with 10% (v/v) fetal bovine serum (FBS) supplemented with penicillin and streptomycin.

Full length of adenoviral E1A gene (NC_001405.1) was cloned into adenoviral shuttle vector pDC315 (Microbix Biosystems Inc.), which named as pDC315-E1A. E1A deletions of amino acids at positions of 30-60 and 120-127 were synthesized by Invitrogen, and also cloned into pDC315 to generate pDC315-mE1A. The plasmid carrying enhanced green fluorescence protein (EGFP), pDC315-EGFP was also constructed and used as a control.

### Adenoviruses construction

The shuttle plasmids pDC315, pDC315-E1A, pDC315-mE1A and pDC315-EGFP were co-transfected into HEK-293 cells with adenoviral cytoskeleton plasmid pBHGE3 (Microbix Biosystems Inc.) using Lipfectamine 2000(Invitrogen) according to manufacturer's instructions respectively. The plaque showed at about 9-14 days after transfection. The transgenes were identified by PCR using specific primers for E1A (mE1A) or EGFP. The adenoviruses Ad-DC315, Ad-DC315-E1A, Ad-DC315-mE1A and Ad-DC315-EGFP (Schematic diagrams were shown in Figure 1B) were grown in HEK293 cells to amplify and titrate by tissue culture infection dose 50 (TCID50) methods. Virus stocks were stored in adenoviral buffer (Ad buffer) (10 mM Tris-HCl, pH 8.0; 2 mM MgCl_2_; 4% sucrose).

### Cell viability assays

Sub-confluent monolayer cultures were trypsinized, and cells were placed on 96-well plates at a density of 10^4^ cells/well. Twenty-four hours later, cells were infected with different adenoviruses at multiple of infection (MOI) of 0, 0.1,1,10 or 100 and cell proliferation was analyzed on day 4 post infection by an MTT-based assay. Cell proliferation was also analyzed on 1, 2, 3, 4 and 5 d after infection at a MOI=10. Briefly, MTT at 0.5 mg/ml was added to the medium in each well, and plates were returned to the incubator for 1 h. The medium MTT was then removed, 500 μl of DMSO were added to each well, and the plate was kept in agitation for 5 min in the dark to dissolve the MTT–formazan crystals. The absorbance of the samples was then recorded at 570 nm. Wells containing medium plus MTT but no cells were used as blanks. Data are the average of three independent experiments performed in triplicate cultures.

### Flow cytometry

Cells were plated in 6-well plates and infected as described and processed at least 48 h later. For cell cycle analysis, cells were trypsinized, washed with PBS, fixed with cold 70% ethanol in PBS at −20°C and extensively washed in cold 1× PBS. The cells were then incubated with 10 μg/ml propidium iodide (PI) and 20 μg/ml RNase for 20 min on darkness. In the case of apoptosis evaluation, the Annexin V-FITC ApoptosisDetection Kit was used as previously described. Briefly, cells were harvested, centrifuged for 5 min at 1200 rpm, washed twice in 1× PBS and resuspended at 10^6^ cells/ml in 1× Annexin binding buffer (Tiangen, Beijing, China). Cells were then stained (5 μlAnnexin V-FITC and 5 μl PI per 100 μl of cell suspension) for 20 min on darkness. Samples were analyzed on a BD FACS Canto II flow cytometer (Becton Dickinson). Data were analyzed by using FACSDiva (Becton Dickinson) and FlowJo (Tree Star Inc.) software packages.

### Immunoblotting analysis

To prepare whole-cell extracts, cells were washed with cold Tris-buffered saline (TBS) and lysed with a RIPA buffer containing 50 mMTris-HCl (pH 7.4), 150 mMNaCl, 2 mM EDTA (pH 8.0), 1% Nonidet P-40, 0.1% SDS, 50 mMNaF, 1 mM Na3VO4, and 1 mM PMSF. Protein concentration was measured by Bradford assay. After SDS-PAGE and semi-dry transfer to polyvinylidenedifluoride membrane (PVDF), the membrane was blocked in a5% non-fat dry milk in TBS with 0.1% Tween 20 for 30 min-1h, and then incubated overnight at 4°C with primary antibodies and for 1h at room temperature with the corresponding fluorescence-labeled secondary antibodies (Sigma). Immunoreactive bands were detected with Odyssey (German). Primary antibodies used for detection were: anti-p53 (DO1, Santa Cruz Biotechnology, USA), anti-mdm2, anti-p21 (Santa Cruz Biotechnology, USA), anti-E1A (clone M73, Merck millipore), anti-Rb, anti-PARP, anti-Bax, anti-Bcl-2, anti-H1 and anti-beta-actin (Abcam, Cambridge, MA, USA).

### Immunoprecipitation

Cells were infected with different Ads for 48h and lysed with RIPA buffer. Cell lysate containing 1 mg protein was added into the tube pre-added 1μg of purified antibody and incubated for overnight at 4°C with gentle rocking. Pre-washed protein-A beads (approximately 12-15μl of a50/50 slurry) were added into the tubes after short spin, and rocked the tubes at 4°C for 30 min. The mix was washed with cold lysis buffer 2-3 times, centrifuged at 2000rpm for 1 min and 20 μl 1X DTT-SDS sample buffer for immunoblotting assay.

### Cellular fractionation

Cells were washed cold PBS and trypsinized with trypsin, and resuspended cell pellet in TM-2 buffer (0.01M Tris-HCl pH7.4, 0.002M MgCl_2_, 0.0005M PMSF) and incubated at room temperature for 1 min and then on ice for 5 min, a final concentration of 0.5% Triton X-100 and incubated on ice for an additional 5 min. The mixed cell suspensions were sheared by several times through a 22-gauage needle and then separated nuclei from the cytosol by centrifugation at 1500RPM, 4°C for 10 min. The supernatant was saved as cell cytosolic fraction. The cellular nuclei pellet was washed with TM-2 buffer twice and resuspended in 100 μl NP-40 buffer (50 mM Tris-HCl pH7.5, 150 mM NaCl, 0.5% NP40, 50 mM NaF, 1 mM NaVO3, 1 mM DTT, 1 mM PMSF and a protease inhibitor cocktail) for incubation 20 min at room temperature with rocking. Separate nuclear fraction from the insoluble by centrifugation at maxi speed at 4°C for 10 min.

### Mouse xenografts

Animal experiment protocols were approved by the Ethics Review Committee for Animal experimentation of China. BALB/C nude mice, male, 4 weeks old, were purchased from the Shanghai SLAC Laboratory Animal Center of Chinese Academy of Sciences (Shanghai, China). HepG2 cells (5×10^6^) were injected subcutaneously into the right flank of nude mice. Viral treatment (at a total dose of 1×10^9^ PFU) was administered for three cycles every 2 days when tumor achieved approximately 5-6 mm in diameter. Phosphate buffered saline (PBS) was used as a control. Tumors were measured every week and their volume was calculated by the formula V=large diameter x (small diameter) ^2^ x 0.5. The survival rate of mice with tumor was assessed until 90 days after first treatment. At the end of the experiment tumors were removed from mice, weighed and immediately fixed in formalin and embedded in paraffin. Tumor sections were cut for further assay.

### Statistical analysis

Data were represented by the mean ± standard deviation (SD). Student's *t* test was used to compare differences between groups. Log-rank test was used to compare differences between groups in the survival rate of mice. Statistical significance was defined as a *P* value less than 0.05.
